# Performance of Large Language Models in Numerical Versus Semantic Medical Knowledge: Cross-Sectional Benchmarking Study on Evidence-Based Questions and Answers

**DOI:** 10.2196/64452

**Published:** 2025-07-14

**Authors:** Eden Avnat, Michal Levy, Daniel Herstain, Elia Yanko, Daniel Ben Joya, Michal Tzuchman Katz, Dafna Eshel, Sahar Laros, Yael Dagan, Shahar Barami, Joseph Mermelstein, Shahar Ovadia, Noam Shomron, Varda Shalev, Raja-Elie E Abdulnour

**Affiliations:** 1 Faculty of Medicine, Tel Aviv University, Chaim Levanon St 55, Tel Aviv, 6997801, Israel, 972 545299622; 2 Kahun Medical Ltd, Givatayim, Israel; 3 Faculty of Medicine, Hebrew University of Jerusalem, Jerusalem, Israel; 4 School of Computer Science and Engineering, The Hebrew University of Jerusalem, Jerusalem, Israel; 5 The Azrieli Faculty of Medicine, Bar-Ilan University, Safed, Israel; 6 Kaplan Medical Center, Rehovot, Israel; 7 Division of Pulmonary and Critical Care Medicine, Department of Medicine, Brigham and Women’s Hospital, Harvard Medical School, Boston, MA, United States

**Keywords:** large language models, questions and answers, dataset, evidence-based medicine, benchmark

## Abstract

**Background:**

Clinical problem-solving requires processing of semantic medical knowledge, such as illness scripts, and numerical medical knowledge of diagnostic tests for evidence-based decision-making. As large language models (LLMs) show promising results in many aspects of language-based clinical practice, their ability to generate nonlanguage evidence-based answers to clinical questions is inherently limited by tokenization.

**Objective:**

This study aimed to evaluate LLMs’ performance on two question types: numeric (correlating findings) and semantic (differentiating entities), while examining differences within and between LLMs in medical aspects and comparing their performance to humans.

**Methods:**

To generate straightforward multichoice questions and answers (Q and As) based on evidence-based medicine (EBM), we used a comprehensive medical knowledge graph (containing data from more than 50,000 peer-reviewed studies) and created the EBM questions and answers (EBMQAs). EBMQA comprises 105,222 Q and As, categorized by medical topics (eg, medical disciplines) and nonmedical topics (eg, question length), and classified into numerical or semantic types. We benchmarked a dataset of 24,000 Q and As on two state-of-the-art LLMs, GPT-4 (OpenAI) and Claude 3 Opus (Anthropic). We evaluated the LLM’s accuracy on semantic and numerical question types and according to sublabeled topics. In addition, we examined the question-answering rate of LLMs by enabling them to choose to abstain from responding to questions. For validation, we compared the results for 100 unrelated numerical EBMQA questions between six human medical experts and the two language models.

**Results:**

In an analysis of 24,542 Q and As, Claude 3 and GPT-4 performed better on semantic Q and As (68.7%, n=1593 and 68.4%, n=1709), respectively. Then on numerical Q and As (61.3%, n=8583 and 56.7%, n=12,038), respectively, with Claude 3 outperforming GPT-4 in numeric accuracy (*P*<.001). A median accuracy gap of 7% (IQR 5%‐10%) was observed between the best and worst sublabels per topic, with different LLMs excelling in different sublabels. Focusing on Medical Discipline sublabels, Claude 3 performed well in neoplastic disorders but struggled with genitourinary disorders (69%, n=676 vs 58%, n=464; *P*<.0001), while GPT-4 excelled in cardiovascular disorders but struggled with neoplastic disorders (60%, n=1076 vs 53%, n=704; *P*=.0002). Furthermore, humans (82.3%, n=82.3) surpassed both Claude 3 (64.3%, n=64.3; *P*<.001) and GPT-4 (55.8%, n=55.8; *P*<.001) in the validation test. Spearman correlation between question-answering and accuracy rate in both Claude 3 and GPT-4 was insignificant (ρ=0.12, *P*=.69; ρ=0.43, *P*=.13).

**Conclusions:**

Both LLMs excelled more in semantic than numerical Q and As, with Claude 3 surpassing GPT-4 in numerical Q and As. However, both LLMs showed inter- and intramodel gaps in different medical aspects and remained inferior to humans. In addition, their ability to respond or abstain from answering a question does not reliably predict how accurately they perform when they do attempt to answer questions. Thus, their medical advice should be addressed carefully.

## Introduction

Clinical problem-solving requires the processing of data using the clinician’s fund of knowledge in the form of illness scripts [1,2], most of which is semantic (differentiating or opting entities). The statistical weight of relationships between data that define an illness is the numerical equivalent of medical knowledge that is essential for prioritizing diagnostic hypotheses and decision-making [3].

Clinicians develop and use numerical knowledge through original research and leverage diagnostic support tools for more complex decision-making [4,5]. However, the explosive amount of medical knowledge and complex health care systems is a tremendous challenge to high-quality, evidence-based medicine (EBM) [6,7].

The breakthrough of large language models (LLMs), which process extensive data and encode knowledge from numerous online studies, shows great promise as tools for medical decision support [8,9]. LLMs provide users with a sense of reliability and accuracy, but evidence shows that they occasionally generate responses that are not based on actual knowledge or give incorrect explanations [10,11]. In addition, their performance on nontextual knowledge, such as medical codes, is limited [12].

Thus, physicians continue to express skepticism regarding LLMs and their capacity to outperform humans [13].

Several benchmark studies have addressed this subject by focusing on lengthy questions from licensing examinations [8,14] or on datasets derived from medical abstracts that could only be answered with “yes,” “no,” or “maybe” [15].

To create a dataset that consists solely of EBM knowledge and is flexible enough to generate both semantic and numeric questions and answers (Q and As), we used the Kahun knowledge graph—a clinically validated artificial intelligence tool that uses a medical, evidence-based knowledge graph. We have developed a methodology to generate Q and As from this knowledge graph and created the EBM question and answer (EBMQA) dataset. The dataset comprises 105,000 short multiple-choice questions based on insights extracted from full-length studies and is aimed to test LLM’s ability to assist physicians.

Finally, we benchmarked two state-of-the-art LLMs: OpenAI’s GPT-4 [16], and Antropic’s Claude 3 Opus (Claude 3) [17], using part of EBMQA. In addition, we compared their results to medical experts. Thus, we evaluated the performance of LLMs in both numerical and semantic Q and A, identified differences within and between LLMs across diverse medical and nonmedical domains, and compared their results to humans. These analyses allowed us to assess whether physicians can trust LLMs.

## Methods

### EBMQA

#### Kahun

Kahun (developed by Kahun Medical Ltd) is a diagnostic tool based on artificial intelligence and structured knowledge graph technologies. The knowledge graph encompasses more than 50,000 peer-reviewed publications and more than 20,000,000 medical relations that were mapped by medical experts [18]. Kahun’s unique structure and its EBM content serve as a reasonable platform to generate the EBMQA. Since the data in EBMQA is based on Kahun’s knowledge graph, which embraces the EBM approach, the gold standard for the answers in the EBMQA is based on published, peer-reviewed medical literature.

#### Questions Structure

All Q and As were derived from Kahun’s knowledge graph. Each question was generated based on data from nodes and edges in the graph and consisted of three main entities: source (usually a disorder related to the target), target (usually a symptom or sign related to the source), and background (usually a relevant population related to the source). In this study, we refer to source, target, or background as entities.

In addition, the relation between entities (derived from data on the edges) determines the question type and the specific template used to generate the questions and the answers. Further explanation regarding template creation is provided in the Multimedia Appendix 1.

**Figure 1.**
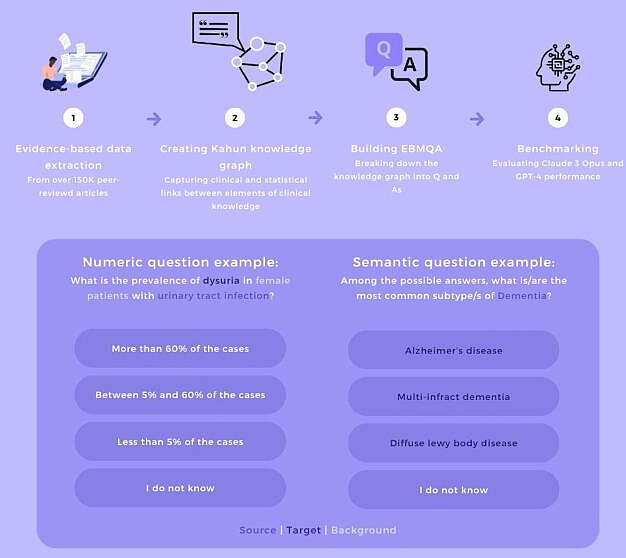
Flowchart of the study: from Kahun's knowledge graph, which references source, target, and background as edges of the graph (1-2), to the evidence-based medicine question and answer dataset and the large language model benchmarking (3-4), which includes both numeric and semantic questions and answers.

EBMQA is comprised of two types of questions: (1) numeric Q and As—derived from connections between a source and a single target. These questions deal with choosing the range in which the correct answer resides and are based solely on this statistical correlation (Figure 1) and (2) semantics Q and As—derived from connections between a source and up to six targets (possible answers). These questions deal with choosing the most common targets related to a source, given a specific relation (eg, subtype, location, and duration), and therefore integrating statistical knowledge across multiple entities and distinguishing between those entities (Figure 1).

Further examples of both numeric and semantic Q and As are provided in Multimedia Appendix 2.

#### Multiple-Choice Question Structure

The questions in the EBMQA are multiple-choice. Numeric Q and As have one correct answer, while semantic Q and As have up to five correct answers. However, for questions in which one does not know the answer, an “I do not know” (IDK) option was added to all questions as a possible answer.

#### Numerical Data and Possible Answers

Each Q and A is based on numerical data derived from Kahun’s knowledge graph, including minimum, maximum, and midvalues, estimating the connections between medical entities. We used statistical methods, including median and median absolute deviation (MAD), to categorize answers into meaningful ranges based on their calculated midvalues. Specific methodologies for categorizing these ranges and detailed statistical information for each Q and A type are provided in Multimedia Appendix 1.

#### Q and A Exclusion

EBMQA aims to provide concise medical Q and As. Therefore, as explained in the “Questions Structure” and “Multiple-Choice Question Structure” sections, questions involving multiple sources, backgrounds, or targets (except semantic questions) were excluded from the EBMQA. This exclusion ensures Q and As with one main, clearly defined subject and a defined target population. In addition, Q and As that are not related to medical knowledge (such as the average length of a season) were removed to maintain focus on medical information. Duplicate questions were excluded, and in such cases, the remaining question retained the average of all duplicated mean values. Therefore, each Q and A in the EBMQA is unique, ensuring no contradictions exist and eliminating any impact duplicates might have on further analysis. To prevent confusion, Q and As with answers indicating “all answers are correct” or “none of the answers are correct” were deleted from the dataset.

#### Labeling

Each Q and A in the study was categorized using multiple medical data labels derived from standardized medical classifications such as those provided by Snomed CT [19] and Kahun’s medical expertise. These classifications include, but are not limited to, medical type, medical subject type, medical discipline, and prevalence. Each Q and A was also analyzed for its question length and distribution of answers. Details on the specific labeling criteria and categories are provided in Multimedia Appendix 1.

### Benchmark Analysis: Q and A Selection and Subanalysis

Due to the relatively different structure of semantic Q and A and the limited number of Q and A, we analyzed all of them separately.

Regarding numeric Q and A types and in the search for meaningful parameters that might influence LLM’s performance, the benchmark included Q and As based on three medical labels (medical subject type, medical discipline, and prevalence) and three nonmedical sublabels (Q and A types, question length, and answers distribution) as further detailed in Multimedia Appendix 3. All Q and As were randomly selected, and although the total number of Q and As per label varied, each label contains an identical number of selected Q and As per sublabeled entity, with no repetition across selections.

### LLMs Prompting

In this study, we used two state-of-the-art LLMs: GPT-4 (gpt-4‐0125-preview) and Claude 3 Opus (claude-3-opus-20240229). Both models’ parameters included temperature=0 and maximum tokens=300. All queries were sent to each LLM using its respective application programming interface. The application programming interface calls were made using R (version 4.2.2; Posit Software, PBC) via R Studio. Further descriptions of the prompts and suitable examples are presented in Multimedia Appendix 1

### Evaluating LLM’s Performance

We evaluated LLM’s performance using the following metrics: (1) accuracy—for both semantic and numeric Q and As, the total number of correct answers suggested by the LLM divided by the total answers suggested by the LLM (excluding IDK answers); (2) answer rate (AR)—for both semantic and numeric Q and As, the total number of both correct and wrong answers suggested by the LLM (excluding IDK answers) divided by the total answers suggested by the LLM (including IDK answers); and (3) majority—for numeric Q and As only, the option that is selected as the correct answer most frequently among all given options in a questionnaire.

### Prompt Sensitivity Analysis

To test both the effects of adding IDK as a possible answer and changing the order of answers (including IDK), 8 different prompts were tested on 100 randomly selected numerical questions. Four prompts included IDK with a different order of possible answers, while four excluded IDK. To prevent bias in the selection process across specific question types, difficulty levels, medical disciplines, and question lengths, and to accurately represent the proportion of each question type in the EBMQA dataset, we randomly selected the questions included in the questionnaire.

### Human Validation

To validate the Q and As in the EBMQA, 2 physicians and 4 clinical-year medical students (3 females and 3 males; aged between 28 and 35 years; all educated and licensed in Israel) answered the questionnaire. Each completed it first with the IDK option and then with mandatory guessing on their previous IDK responses. Their accuracies, with and without guessing, were compared to LLM’s performance.

### Analysis and Variables

All statistical analyses were performed using R Studio (R version 4.2.2). Categorical variables were represented as percentages, while continuous variables were represented as means and SDs for normally distributed data, or medians and IQRs otherwise. The cutoff for statistically significant results was set at *α*=.05, and 95% CIs were calculated. Proportions comparison was conducted using the “Proportion test.” Spearman correlation was used to analyze correlations between 2 quantitative variables.

### Ethical Considerations

This study was approved by the Tel Aviv University Ethics Committee (institutional review board protocol number 0008527‐2). All questionnaire data were anonymized and deidentified in accordance with HIPAA (Health Insurance Portability and Accountability Act) Safe Harbor privacy rules. Informed consent was received by all the participants. Before answering the questionnaire, participants were informed that the study was being conducted for research purposes only, no personal or sensitive data would be collected, answers would not be identified in the results, participation would be voluntary, and informed consent would be provided by answering the questionnaire. Appropriate measures were taken to ensure compliance with relevant privacy guidelines. No compensation was provided to participants.

## Results

### EBMQA

The EBMQA contains 105,222 Q and As. In addition, each Q and A pair was labeled according to metadata labels and medical labels.

#### Medical Labels

The EBMQA encompasses diverse medical data types, including a unique count of 7746 “Disorders,” 2547 “Signs or Symptoms,” 1243 “Lab tests,” 885 “Imaging or procedures,” 474 “Background” data (demographics, habits, family history, etc), and more (Multimedia Appendix 4).

Among the medical subject types, “Disorders” was the most abundant with 45,964 Q and As, followed by “Symptoms and Signs” with 30,152 Q and As, “Lab test” with 5966 Q and As, and “Imaging or Procedures” with 4374 Q and As. All the other subjects encompass 640 Q and As (Multimedia Appendix 4).

Focusing on medical discipline, the EBMQA contains 64,846 relevant Q and As: the leading medical discipline was the digestive system with 9879 Q and As, followed by the cardiovascular system with 7847 Q and As, and infectious diseases with 7798 Q and As. The musculoskeletal system had the least number of Q and As, that is, 2832 (Multimedia Appendix 4).

Regarding the “Prevalence” label, the median prevalence was 1e-4 (IQR 2e-6 to 1.98e-4) and the MAD was 9.810102e-05 (IQR 1.9e-6 to 1.98e-4). Of these, 36,653 Q and As focused on high-prevalence disorders, 22,139 Q and As focused on moderate-prevalence disorders, and 2531 Q and As focused on low-prevalence disorders (Multimedia Appendix 4).

#### Metadata Labels

EBMQA includes 13 distinct Q and A types (Multimedia Appendix 4). The most frequent Q and A type was “Sensitivity” with 70% (74,140/105,222) of the total Q and As. Eight Q and A types had less than 900 Q and As: specificity, positive likelihood ratio, negative likelihood ratio, relative risk, prevalence, positive predictive value, negative predictive value, and associated risk.

In total, the median number of words per question (including the question, instructions, and possible answers) in the EBMQA was 57 (IQR 53‐66), with a MAD of 5. The medium-length question group had the majority of Q and As (ie, 59,998), whereas the short-length question group had the fewest Q and As (ie, 9968) (Multimedia Appendix 4). Focusing on each Q and A type, “Risk Factor” Q and As had the longest median of question length with 81 (IQR 80‐84) words, while “Sensitivity” Q and As had the shortest with 54 (IQR 52‐58) words (Multimedia Appendix 5).

Regarding numeric questions with 3 range values, the most frequently distributed answer was the midrange values (46,431 Q and As), followed by the low-range values (28,598 Q and As), and the high-range values (14,292 Q and As; Multimedia Appendix 6).

### Benchmark Analysis

Of the 105,222 Q and As, a set of 24,542 questions was presented to each LLM. “Numeric” Q and As comprised 90% (22,000/24,542) of the questions, whereas “semantic” Q and As accounted for the remaining 10% (2542/24,254).

Both LLMs demonstrated better performances in the semantic Q and As than in the numeric Q and As in terms of accuracy (Claude 3: 68.65%, 1592.78/2320, vs 61.29%, 8583/14,005, *P*<.001; GPT-4: 68.38%, 1708.85/2499, vs 56.74%, 12,038/21,215, *P*<.001) and AR (Claude 3: 94.62%, 2320/2542, vs 63.66%, 14,005/22,000, *P*<.001; GPT-4: 98.31%, 2499/2542, vs 96.4%, 21,215/22,000, *P*<.001).

From an intermodel perspective, Claude 3 outperformed GPT-4 in numeric accuracy, though no significant difference was found in semantic accuracy. However, in comparison to Claude 3, GPT-4 had a higher AR in both semantic and numeric questions (Table 1). Focusing on numeric accuracy and excluding any questions that one or both LLMs responded to with IDK results in the exclusion of 8133 questions and a total of 13,867 answered questions. Keeping the same trend, Claude 3 outperformed GPT-4 in numeric accuracy (8491/13,867 vs 8255/13,867; *P*=.004).

**Table 1.** Claude 3 versus GPT-4: overall accuracy and answer rate for semantic and numeric questions.

Question type and model	Accuracy, % (n/N)	Answer rate, % (n/N)	Proportion test, *P* value
Semantic			.86
Claude 3 (Anthropic)	68.65% (1592.78/2320)	94.62% (2320/2542)	
GPT-4 (OpenAI)	68.38% (1708.85/2499)	98.31% (2499/2542)	
Numeric			<.00001
Claude 3	61.29% (8583/14,005)	56.74% (12,038/21,215)	
GPT-4	63.66% (14,005/22,000)	96.4% (21,215/22,000)	

### Prompt Sensitivity Analysis

Based on the results of the questionnaire, the average accuracy of Claude 3 with the IDK option versus without it was not significantly different (64.25%; mean 64.25, SD 3.95, vs 59.25%; mean 59.25, SD 5; *P=.*17). A similar trend was noted for GPT-4 (55.75%; mean 55.75, SD 1.71, vs 53.25%; mean 53.25, SD 2.89; *P=*.24). In addition, within each subgroup—Claude 3 with and without the IDK option, and GPT-4 with and without the IDK option—no single answer-option-order prompt was significantly superior to the others (MultimediaAppendices 7  8).

### Human Validation

Claude 3 and GPT-4 achieved higher average accuracy rates, with or without the IDK option, than random guessing (33%, n=33) or majority guessing (47%, n=47). However, both models had lower average accuracy rates compared to humans with the IDK option (82.3%; mean 82.3, SD 2.82) or without it (78.2%; mean 78.2, SD 3.6; Multimedia Appendix 9).

### Numeric Q and A Subanalysis

The accuracy gap between the highest and lowest accuracy rates in each LLM was calculated, revealing a median difference of 7% (IQR 5%‐10%; Multimedia Appendix 10). Focusing on disorders selected sublabels, Claude 3 performed well in neoplastic disorders but struggled with genitourinary disorders (69%, 676/984 vs 58%, 464/803; *P<*.0001), while GPT-4 excelled in cardiovascular disorders but struggled with neoplastic disorders (60%, 1076/1783 vs 53%, 704/1316; *P=*.0002; Multimedia Appendix 11). Furthermore, among sublabel disorders queried over 200 times, Spearman correlations between Q and A and accuracy rate in both Claude 3 and GPT-4 were insignificant (*ρ=0.12, P=*.69*; ρ=0.43, P=*.13).

## Discussion

### Principal Findings

This study aimed to highlight the current gaps in the medical knowledge of LLMs and their current ability to surpass humans. We presented a method to create an EBMQA from a structured knowledge graph and benchmarked two state-of-the-art LLMs (GPT-4 and Claude 3) [16,17]. We demonstrated that both LLMs performed better in semantic Q and As than in numerical Q and As by asking more than 24,000 Q and As (Table 1). Claude 3 outperformed GPT-4 in numerical Q and As and showed similar results in semantic Q and As, although it exhibited significantly lower ARs (Table 1). A validation test indicated that the numerical accuracy rates of Claude 3 and GPT-4 were higher than majority guessing but remained lower than those of medical experts (Multimedia Appendix 12).

### Prior Work and Novel Contribution

The use of knowledge graphs for evaluating LLMs is gaining popularity [20-22]. Kahun’s structured knowledge graph enabled us to generate both semantic and numeric labeled Q and A pairs, without using advanced models [22]. Our Q and A generation process, which relies on templates designed to fit a source-target-background graph structure, can apply to other graphs with a similar structure. In addition, this relatively large knowledge graph allowed us to create a massive EBM dataset. Moreover, we embraced a data-driven approach in which distractors were based on subanalysis distribution rather than specific or random values.

The EBMQA, which consists of 105,222 straightforward single-line Q and As, was designed to mimic physicians’ strategy of breaking complex medical scenarios into less complicated problems, unlike medical licensing examination datasets, which are typically complex-case oriented [14,23]. In addition, the EBMQA addresses numeric and semantic data, which is considered fundamental for physicians [24,25], while dealing with data from studies and embracing the EBM approach [7], as opposed to the abstracted-based yes or no or maybe Q and As in PubMedQA [15].

A major concern regarding applying LLMs in health care is the uncertainty of providing solid evidence that supports their answers [8]. Clinical evidence predominantly relies on statistical and numerical data. Thus, it is imperative to examine whether LLMs can deliver this type of reasoning. It has been shown that LLMs are more capable when given semantic questions rather than numerical questions, though in a relatively small sample size (smaller than 200 Q and As) [26]. As far as we know, we were the first to show this trend in the medical field while using a much larger scale (Table 1). Furthermore, since both semantic and numeric questions in the EBMQA may address the same entities but from different perspectives, our study questioned whether LLMs can support their semantic answers with statistical data.

In addition, as LLMs are gaining more popularity as decision-support tools [8,9], understanding which types of questions will yield more precise answers, as demonstrated in our semantic and numeric analysis, could benefit not only the medical community but also the general use of LLMs.

A recent benchmark analysis, focused on nephrology Q and As, only found that GPT-4 outperformed Claude 2 [27]. Although our intermodel examination did not include a direct nephrology compression due to a different classification method, it reveals that generally Claude 3 outperformed GPT-4, and specifically in a variety of medical disciplines such as neoplastic disorders, nervous system, and more. Our results raise the need to constantly benchmark new LLMs as they continuously improve.

Regarding internal model variations, the differences in accuracy between the highest and lowest performing medical disciplines, 8% for Claude 3 and 6% for GPT-4, support previous benchmarks that found LLM performance can vary across different medical disciplines [27,28].

Moreover, this comprehensive benchmark widens the medical scope and further supports both intra- and inter-model differences by exploring medical subjects: Claude 3 favors “Imaging and Procedures” and struggles with “Disorders” (64%, 463/719 vs 60%, 3181/5296; *P*=.03, respectively), while GPT-4 excels in “Imaging and Procedures” but struggles with “Lab tests” (60%, 1017/1683 vs 53%, 1008/1886; *P*<.0001, respectively). Thus, our study underscores two vital, yet distinct, aspects in the integration of LLMs into daily medical practice: first, the specific areas of medical expertise where each LLM excels, and second, comparing which LLM is superior within each area of medical expertise.

As suggested by previous studies, LLMs are highly sensitive to question wording, structure, and subject matter. Consequently, direct comparisons across different benchmarks, which rely on distinct datasets, may yield varying scores that do not necessarily reflect a genuine knowledge gap but rather other confounding factors such as those mentioned at the start of this section [29,30]. For example, Katz et al [28] reported that GPT-4’s accuracy rates ranged from 17.42% (n=21) to 74.7% (n=90) across various medical disciplines. In contrast, our study found GPT-4’s accuracy rates ranged more narrowly, from 53.5% (n=704) to 60.35% (n=1076). This discrepancy could be partially attributed to the differing medical disciplines emphasized in each study, as well as variations in question structure. While Katz et al [28] used five different exams with potentially diverse question formats, all questions in our study were generated using the same templates, resulting in a relatively narrower accuracy range. Furthermore, Liu et al [31] benchmarked GPT-4 and Claude 3 using the Japanese National Medical Examination and reported accuracy rates of 80.0% (n=720) and 83.6% (n=752), respectively. Although our study observed a similar trend, the accuracy rates differed, with 56.74% (n=12,038) for GPT-4 and 61.29% (n=8583) for Claude 3. A key distinction between the 2 benchmark studies lies in the question structure: Liu et al’s dataset included questions with multiple correct answers, whereas our numerical questions had only a single correct answer. These examples highlight both the importance of treating direct comparisons among LLM benchmark studies with caution and the value of developing multiple high-quality benchmarks on unique, well-designed datasets.

### Clinical Impact and Further Needed Research

As the debate over whether models surpass humans persists [27,28], the outcomes of our validation tests suggest that humans still excel in certain medical tasks. Therefore, we support further evaluations of LLMs before using them in medical settings.

Furthermore, the insignificant correlation between accuracy and AR contradicts the theory that a model’s confidence in its response reflects its subject expertise [32]. Thus, abstaining from providing an answer failed to explain the intramodel variance results, specifically across medical disciplines. Notably, recent research has shown that LLMs exhibit varied abstention abilities, which is consistent with our finding and may be influenced by model-specific characteristics, context nature, and question type [33]. For instance, some LLMs find it challenging to abstain from Boolean questions with standard prompts. Intriguingly, modifying the context by introducing irrelevant information can occasionally enhance abstention performance and, thereby, improve overall task accuracy [33].

This evidence, along with our findings, raises concerns that without prior knowledge of both the medical field and the model, the trustworthiness of LLMs is questionable.

In terms of prompt engineering, our sensitivity analysis showed relatively small SDs in prompt accuracy, which supported our prompt stability. In addition, although insignificant, the IDK prompt yielded higher accuracy and was therefore used. Moreover, changing the order of the distractors did not significantly affect the LLM’s performance.

### Limitations

Our benchmark has several limitations. First, although medically tuned LLMs have shown promising results [34], they are not publicly available and hence were not included in this study. We highly recommend conducting a similar benchmark that includes these LLMs. Second, we did not use additional context for the prompt or use external methods such as retrieval-augmented generation, which could potentially improve the results. We chose not to use these methods because we believe that, currently, physicians are asking LLMs straightforward questions. In addition, some of these external methods are not widely accessible to end users and are far more complex than the typical daily use of LLMs that we aimed to replicate. Given that these methods might influence the results, we strongly recommend conducting research that focuses on retrieval-augmented generation or providing extra context in the prompt. Third, the study was designed so that the models and human participants would only choose one suggested answer, without providing additional information or feedback. Therefore, we support further studies to examine these responses by the models, while considering feedback from human physicians. Fourth, this study did not include a subanalysis regarding progressive patterns such as the abstention behavior. Therefore, we recommend further research on the subject. Another limitation is the known potential biases when using LLMs, such as the training on an enormous amount of data, which may include bias and inaccuracies itself, or may also harm the contextual understanding of medical cases and result in poorer answers due to undertraining on less common medical disciplines [35].

### Conclusions

On the EBMQA dataset, which resembles physicians’ problem-solving approach, LLMs were better at solving semantic than numeric questions. Despite Claude 3 surpassing GPT-4, both LLMs exhibited inter- and intramodel gaps in medical knowledge. In addition, human participants outperformed both LLMs on numeric questions. These results suggest that LLMs’ responses, especially numeric ones, should be considered cautiously in clinical settings.

## Supplementary material

10.2196/64452 Multimedia Appendix 1 Additional data and explanations.

10.2196/64452 Multimedia Appendix 2 Examples for semantic and numeric questions and answers.

10.2196/64452 Multimedia Appendix 3 Numeric question and answers benchmark subanalysis according to medical and nonmedical labels and sublabels.

10.2196/64452 Multimedia Appendix 4 Distribution of the data and labels in the evidence-based medicine questions and answers: (A) unique medical data type, (B) question subject, (C) medical discipline, (D) disorders prevalence, (E) question type, and (F) question length.

10.2196/64452 Multimedia Appendix 5 Distributions of answers according to question type.

10.2196/64452 Multimedia Appendix 6 Distribution of the correct answer with midvalues ranging from 0 to 1 of the questions and answers in the evidence-based medicine question and answer dataset, categorized by the overall median value of each question and answer type and the corresponding median absolute deviation: 0≤ midvalue < overall median - median absolute deviation (short), overall median - median absolute deviation ≤ mid value ≤ overall median + median absolute deviation (medium), and overall median + median absolute deviation ≤ mid value <1 (long).

10.2196/64452 Multimedia Appendix 7 Sensitivity analysis of four prompts with the “I do not know” option was assessed according to their accuracy. Each row represents a different order of the possible answers. The order of the possible answers in the prompt is based on the sequence of letters or symbols, separated by hyphens, from left to right. Each letter or symbol represents a frequency range determined by the relevant overall median and the median absolute deviation: frequency range ≥ overall median + median absolute deviation (frequent [F]), overall median - median absolute deviation ≤ frequency range ≤ overall median + median absolute deviation (medium [M]), frequency range ≤ overall median - median absolute deviation (rare [R]), and I do not know.

10.2196/64452 Multimedia Appendix 8 Sensitivity analysis of four prompts without the “I do not know” option was assessed according to their accuracy. Each row represents a different order of the possible answers. The order of the possible answers in the prompt is based on the sequence of letters or symbols, separated by hyphens, from left to right. Each letter or symbol represents a frequency range determined by the relevant overall median and the median absolute deviation: frequency range ≥ overall median + median absolute deviation (frequent [F]), overall median - median absolute deviation ≤ frequency range ≤ overall median + median absolute deviation (medium [M]), frequency range ≤ overall median - median absolute deviation (rare [R]).

10.2196/64452 Multimedia Appendix 9 Human and prompt validation.

10.2196/64452 Multimedia Appendix 10 Numeric question and answer accuracy rate sublabel analysis: (A) answer distribution, (B) medical discipline, (C) medical subject type, (D) question and answer type, (E) disorder prevalence, and (F) question length. Red asterisks represent proportion *P* values: .05< *<.01, ***<.0001.

10.2196/64452 Multimedia Appendix 11 Proportion comparison according to sublabels.

10.2196/64452 Multimedia Appendix 12 Validation test: each large language model was tested 8 times—4 times with the “I do not know” (abstain) option, using the same prompt but in different order of possible answers, and 4 times without the abstain option. In addition, 6 medical experts were tested: first with the abstain option, and then without. Error bars indicating 1 SD and answer rate bars were added only for trials with the abstain option.

## Abbreviations

AR: answer rate
EBM: evidence-based medicine
EBMQA: evidence-based medicine question and answer
HIPAA: Health Insurance Portability and Accountability Act
IDK: I do not know
LLM: large language model
MAD: median absolute deviation
Q and A: question and answer
