# Supplementary material for: Performance of Large Language Models in Numerical Versus Semantic Medical Knowledge: Cross-Sectional Benchmarking Study on Evidence-Based Questions and Answers
Source: J Med Internet Res. 2025 Jul 14;27:e64452. doi: 10.2196/64452 (PMC12279315; doi:10.2196/64452)
Supplement: Multimedia Appendix 1 [file jmir-v27-e64452-s001.docx]

# Appendix:

## EBMQA:

### Template creation:

Kahun's knowledge graph contains nodes, connected through edges. Each evidence-based knowledge (from an article) is represented with two nodes and one edge. For example, node1 contains data regarding a source (for example, a disorder) and its background (a population). Node1 is connected, through edge1, to node2, which contains data regarding a target (for example, a symptom). Edge1 contains data such as the type of connection between node1 and node2, the numerical value of the connection and additional information (for example, a prevalence of 60%). For each connection in the graph, a specific template with placeholders for the data from node1 and node2 was designed. As shown in Figure 1, for the "prevalence" type of connection, the template was: "What is the prevalence of *target* in *background* with *source*?". Using this method and the structure of the graph, we were able to create the EBMQA.

### Numerical data and possible answers:

Since each QA is based on data stored in Kahun's knowledge graph, all the possible answers (correct or wrong) in the semantic QA and the correct answer in the numeric QA are backed with numerical data and citations. Therefore, “wrong” answers in the semantic QA represent connections that exist in the knowledge graph.

The data behind each possible answers (both correct and wrong) in the semantic QA and behind each correct answer in the numeric QA includes the following numerical values:

- Minimum value - A lower estimation of the connection between the source and the target given the concept.
- Maximum value - A higher estimation of the connection between the source and the target given the concept.
- Mid value - The Mean of the "Minimum value " and the "Maximum value".

In a numeric QA we used the median of all the mid values (overall median) which are related to the specific numeric QA type. We calculated the median absolute deviation (MAD) to form ranges of answers as explained below. The correct answer is a range that includes the Mid value.

For questions with mid values ranging from 0-1 (such as Prevalence, Association, etc.), four possible answers were defined by a single MAD from the overall median: from 0 to *median - MAD* (1st answer- low range values), from *median - MAD* to *median + MAD*, (2nd answer- mid range values), greater than *median + MAD (*3rd answer- high range values) and an IDK answer.

For QA types with Mid values that can be greater than 1 (such as Relative Risk, Odds Ratio, etc.), a value smaller than 1 indicates that the risk of the outcome is decreased by the exposure and a value greater than 1 indicates that the risk of the outcome is increased by the exposure. Therefore, we separated decreasing and increasing values and evaluated the median of values between 0-1 and the median of values greater than 1. As a result, those QA types have five possible answers: from 0 to the decreasing median, from the decreasing median to 1, from 1 to the increasing median, greater than the increasing median and an IDK answer.

On the contrary, a semantic QA can have one or more correct answers out of the possible answers:

- A question will have one correct answer if the highest mean value of all possible answers is at least 10% higher than the following highest mean value.
- A question will have more than one answer if there are several answers with mean values that have less than a 10% difference between them and the highest mean value.
- If all the possible answers have less than a 10% difference between them, all answers are considered correct.

### Labeling:

Each QA was classified according to the following medical data labels:

1. Medical type - each entity (in the question) was classified according to groups of clinical aspects as suggested by Snomed CT (a medical clinical terminology database used by the U.S. Federal Government) [<https://www.nlm.nih.gov/healthit/snomedct/index.html> ] or by Kahun’s medical team. For example: Disorders, Symptoms or Signs, Lab tests etc.
2. Medical subject type – the label of the “Medical type” of the subject in the question, usually the target’s “Medical type”. Relevant to all types of QAs except form “Association”.
3. Medical Discipline - entities were classified according to groups of medical topics as suggested by Snomed CT [<https://www.nlm.nih.gov/healthit/snomedct/index.html> ] or by Kahun’s medical team. For example: Respiratory system, Neurological system, Cardiovascular system etc.
4. Unique Medical Discipline - the name of the unique Medical Discipline in the question. This label is relevant only if there is one type of Medical Discipline in the question.
5. Prevalence - the prevalence of the disorder in the QA as dated in Kahun’s knowledge graph. This label is relevant only if the question includes one disorder, as determined by having a single “Disorder” Medical type label. Inspired by the Head-Torso-Tail approach [20], we developed a classification method for disease prevalence. QAs were categorized into high, moderate and low prevalence disorders based on the overall median prevalence of QAs that have only one disorder. Widespread disorders have a prevalence higher than the *median + MAD*, rare disorders have a prevalence lower than the *median - MAD*, and common disorders fall in between these thresholds.

Additionally, each QA was classified according to two main metadata labels:

1. QA type - based on the data from Kahun’s knowledge graph.
2. Question length - QAs were categorized into long, medium, and short length based on the overall median question length. A long question length is longer than the *median + MAD* value, a short question length is shorter than the *median - MAD*, and medium question length falls in between these thresholds.
3. Answers distribution- Relevant only for QAs with mid values ranging from 0-1 and including three answers: the 1st answer- low range values, the 2nd answer- mid range values and the 3rd answer- high range values.

## Benchmark Analysis:

### QA selection:

Detailed descriptions of the QAs in the benchmark (Figure S1):

1. Medical subject type (medical): four medical subject types were selected, each with 1,000 QAs per type: “Disorders”, “Signs or Symptoms”, “Imaging or Procedures” and “Lab Test”.
2. Medical Discipline (medical): seven medical discipline types were selected, each with 500 QAs per type: Digestive system, Cardiovascular system, Infectious disease, Nervous system, Respiratory system, Genitourinary system and Neoplastic disease.
3. Prevalence (medical): An equal number of 1,500 QAs were selected from each of the three “Prevalence” categories.
4. QA types (non-medical): four QA types were selected, each with 1,000 QAs per type: “Sensitivity”, ”Risk Factor”, “Association” and “Incidence”.
5. Question length (non-medical): An equal number of 1,000 QAs were selected from each of the three “Question length” categories.
6. Answers distribution (non-medical): An equal number of 1,000 QAs were selected from each of the three “Answers distribution” categories.

### LLMs prompting:

The prompt for each QA included the question itself, a specific text asking to choose the answer only from the provided possible answers while not adding additional text. Moreover, an IDK option was added to the possible answers.
An example for the semantic prompt:

Among the possible answers, What is/are the most common subtype/s of Dementia? The possible answers are: ' Alzheimer's disease ' , ' Multi-infarct dementia ' , ' Diffuse Lewy body disease ' , ' Frontotemporal dementia ' . You must base your response exclusively on the possible answers provided. No other words or answers are allowed. You can choose a single answer or multiple answers from the answers provided. If you do not know the answer to the question, respond with ‘I do not know’.

An example for the numeric prompt:

What is the prevalence of dysuria in female patients? Choose the correct answer from the following options, without adding further text: (1) Greater than 54%, (2) Between 5% and 54%, (3) Less than 5%, (4) I do not know (only if you do not know what the answer is).
