# Supplementary material for: Performance of Large Language Models in Numerical Versus Semantic Medical Knowledge: Cross-Sectional Benchmarking Study on Evidence-Based Questions and Answers
Source: J Med Internet Res. 2025 Jul 14;27:e64452. doi: 10.2196/64452 (PMC12279315; doi:10.2196/64452)
Supplement: Multimedia Appendix 2 [file jmir-v27-e64452-s002.docx]

### Table S1 - Examples for semantic and numeric QAs

| Question type | Question | Possible Answers |
| --- | --- | --- |
| Semantic | Which biological sex has an increased likelihood of Osteoarthritis within the general population? | - Male - Female - I do not know |
| Semantic | Which age group/s is/are the most commonly associated with Crohn's disease? | - 60-90 Years - 20-29 Years - I do not know |
| Semantic | What is/are the most common location/s of Patch in patients with Mycosis fungoides? | - Breast part - Buttock structure - Lower trunk - Skin structure of inguinal region  - I do not know |
| Numeric | How does Oral contraception influence the chance of Cerebral venous sinus thrombosis? | - Increases the chance by greater than 2.5 times - Increases the chance between 1.01 and 2.5 times  - Decreases the chance between 0.7 and 0.99 times - Decreases the chance by less than 0.7 times - I do not know |
| Numeric | What is the positive likelihood ratio of dyspnea at rest in patients with Asthma? | - Greater than 3.7 - Between 1.01 and 3.7 - Between 0.35 and 0.99 - Less than 0.35  - I do not know |
| Numeric | Is the association between Factor V deficiency and Cerebral venous thrombosis low, medium or high? | - High (greater than 42% of the cases) - Medium (between 5% and 42% of the cases) - Low (less than 5% of the cases) - I do not know (only if you do not know what the answer is) |
