# Supplementary figures and images for: Performance of Large Language Models in Numerical Versus Semantic Medical Knowledge: Cross-Sectional Benchmarking Study on Evidence-Based Questions and Answers
Source: J Med Internet Res. 2025 Jul 14;27:e64452. doi: 10.2196/64452 (PMC12279315; doi:10.2196/64452)

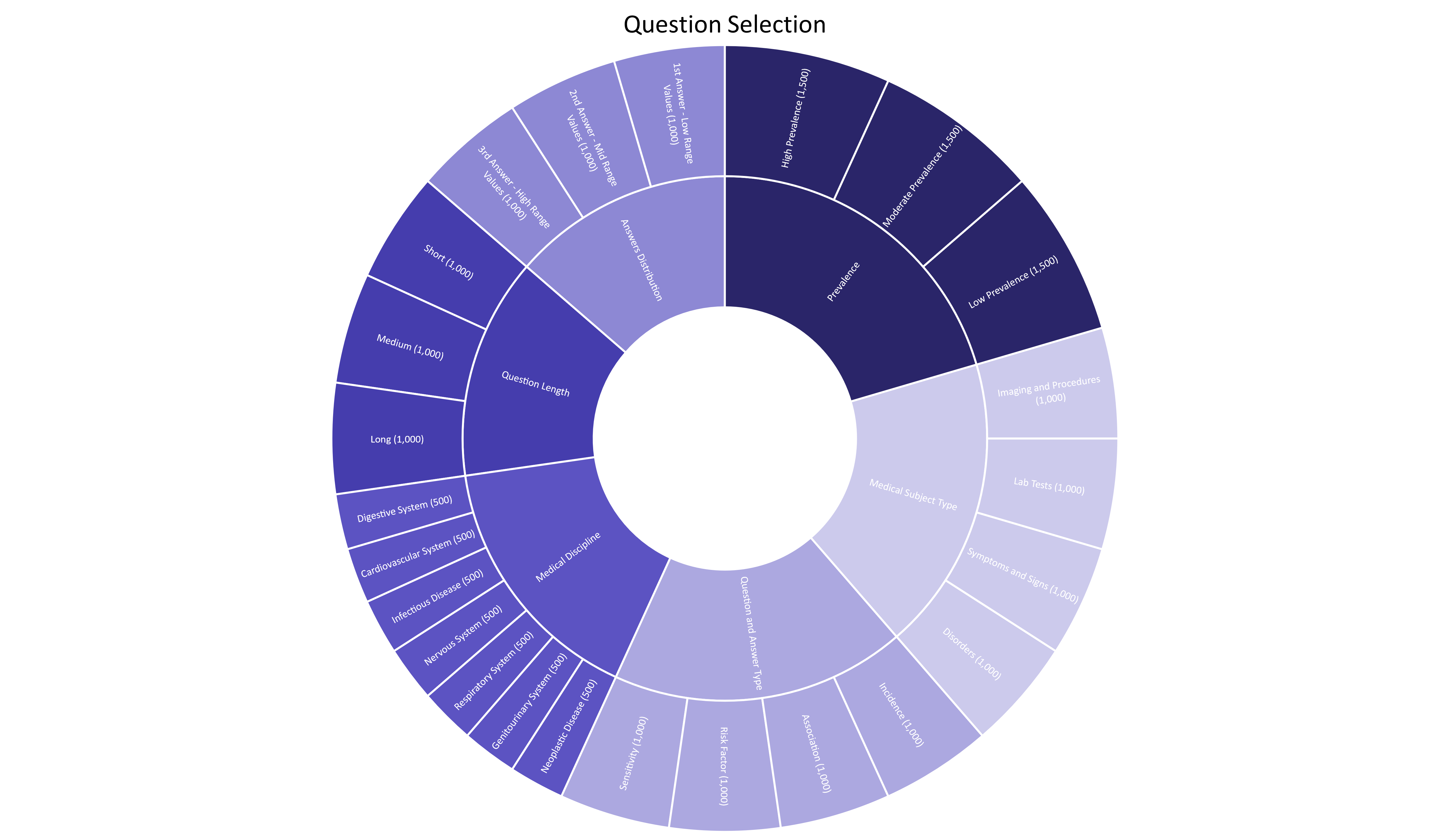

Supplement: Multimedia Appendix 3 [file jmir-v27-e64452-s003.png]

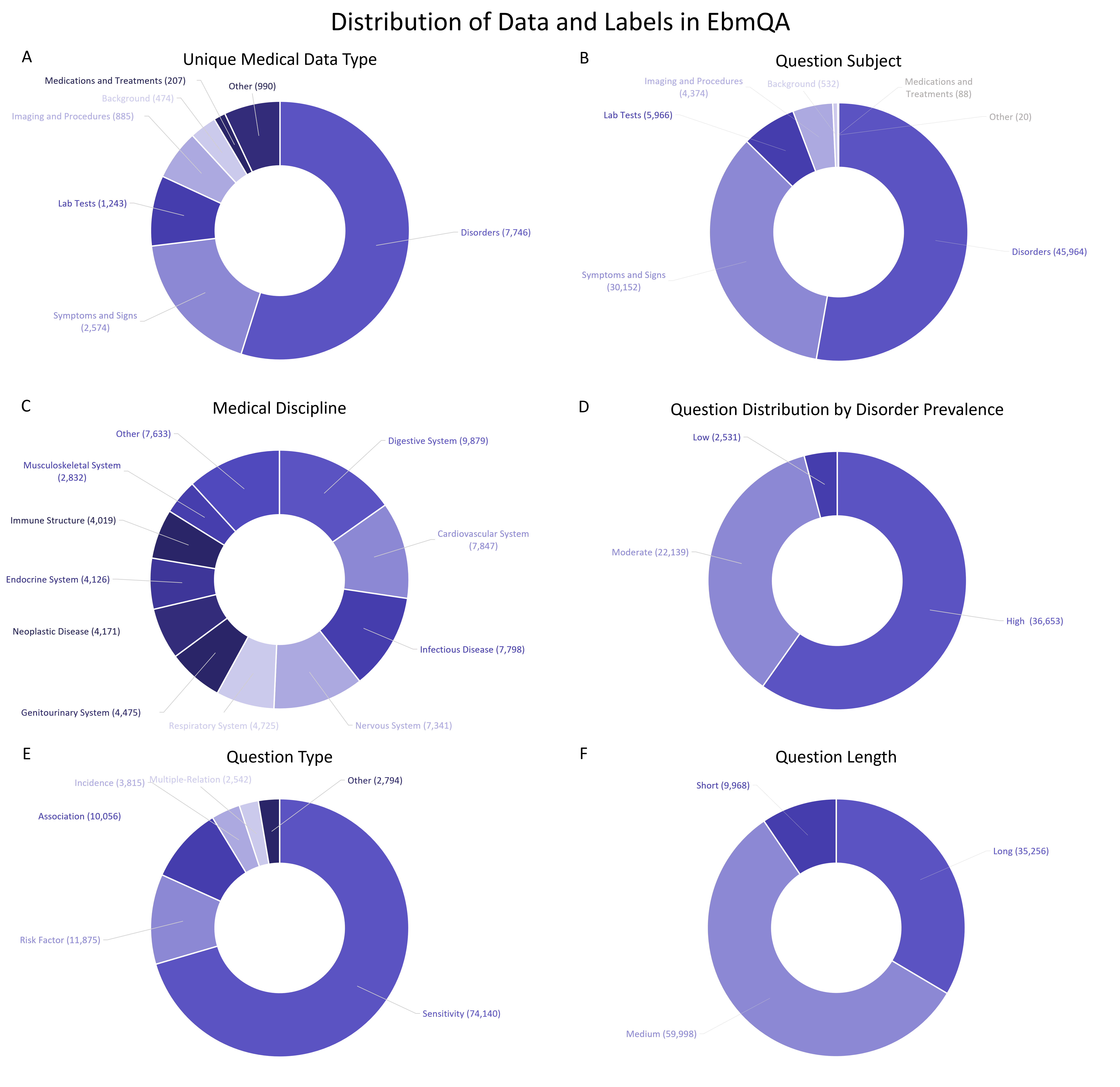

Supplement: Multimedia Appendix 4 [file jmir-v27-e64452-s004.png]

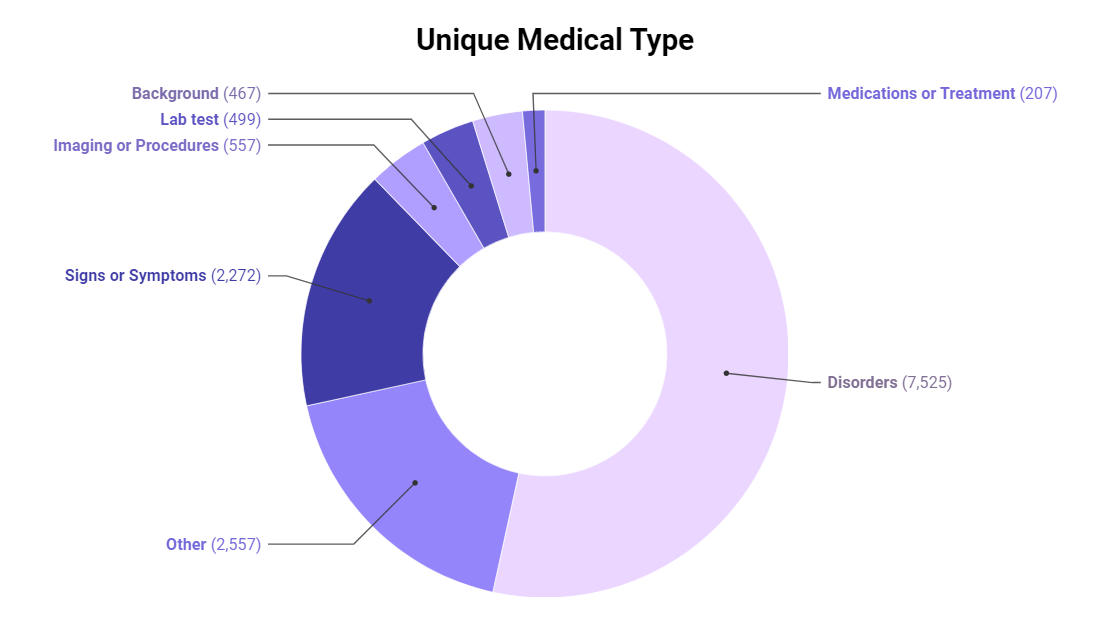

Supplement: Multimedia Appendix 6 [file jmir-v27-e64452-s006.png]

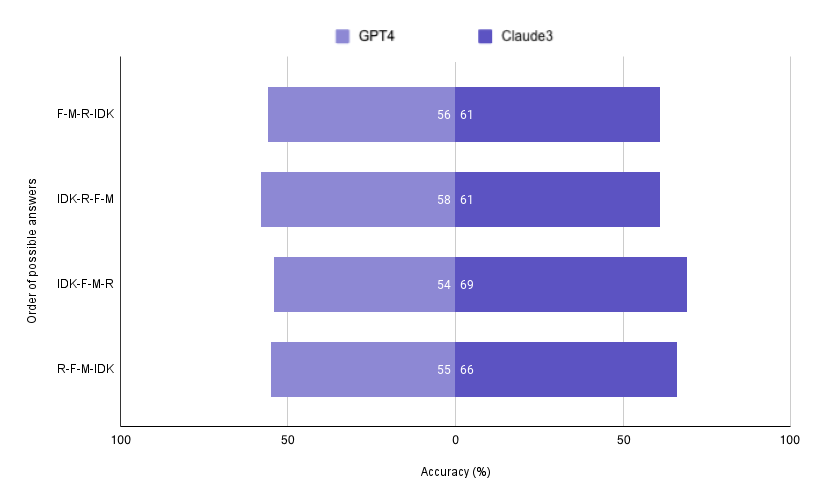

Supplement: Multimedia Appendix 7 [file jmir-v27-e64452-s007.png]

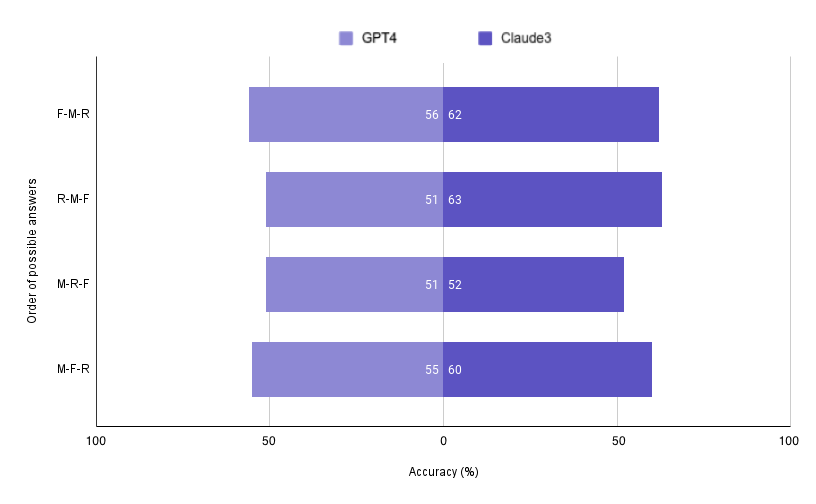

Supplement: Multimedia Appendix 8 [file jmir-v27-e64452-s008.png]

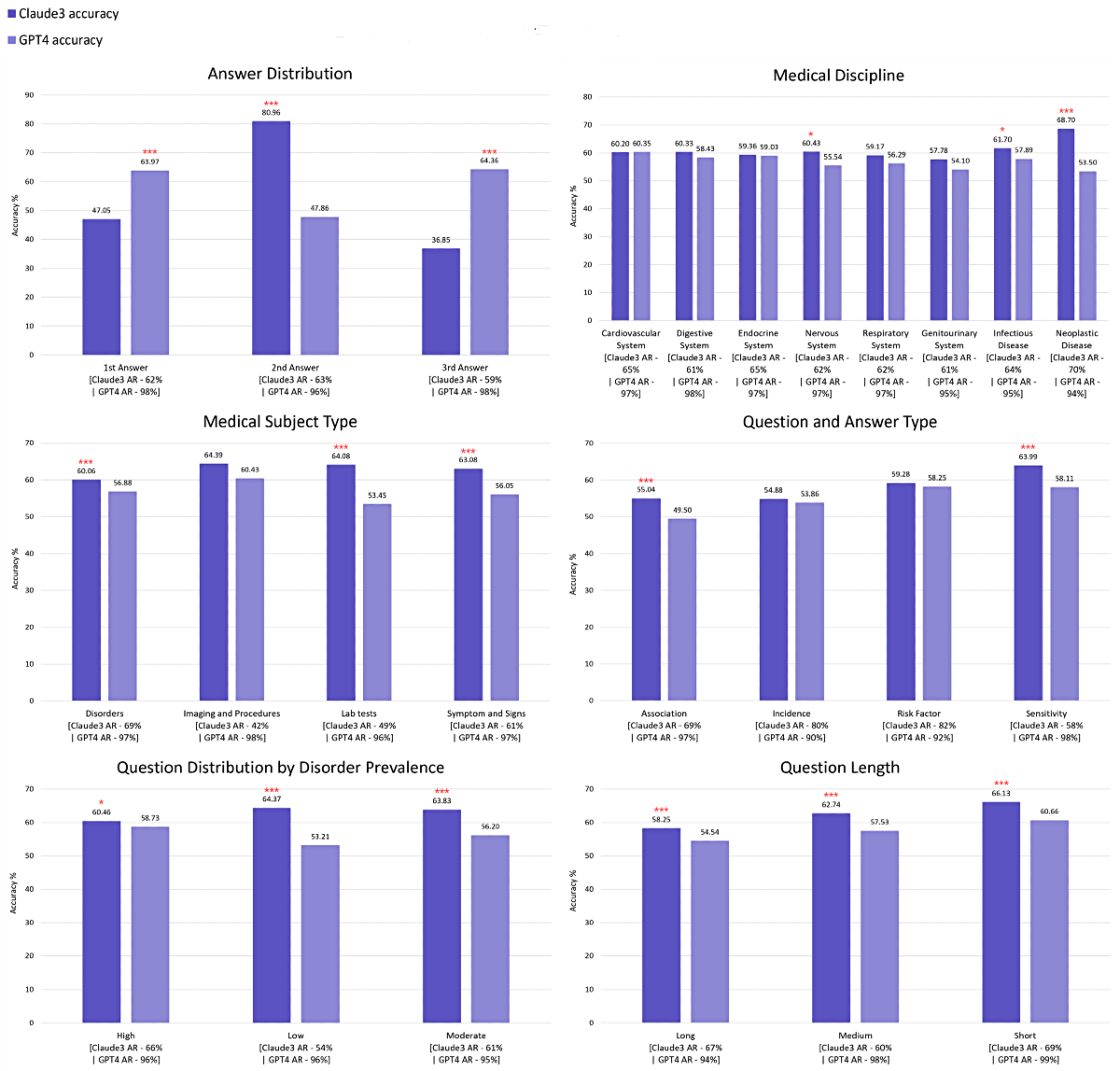

Supplement: Multimedia Appendix 10 [file jmir-v27-e64452-s010.png]

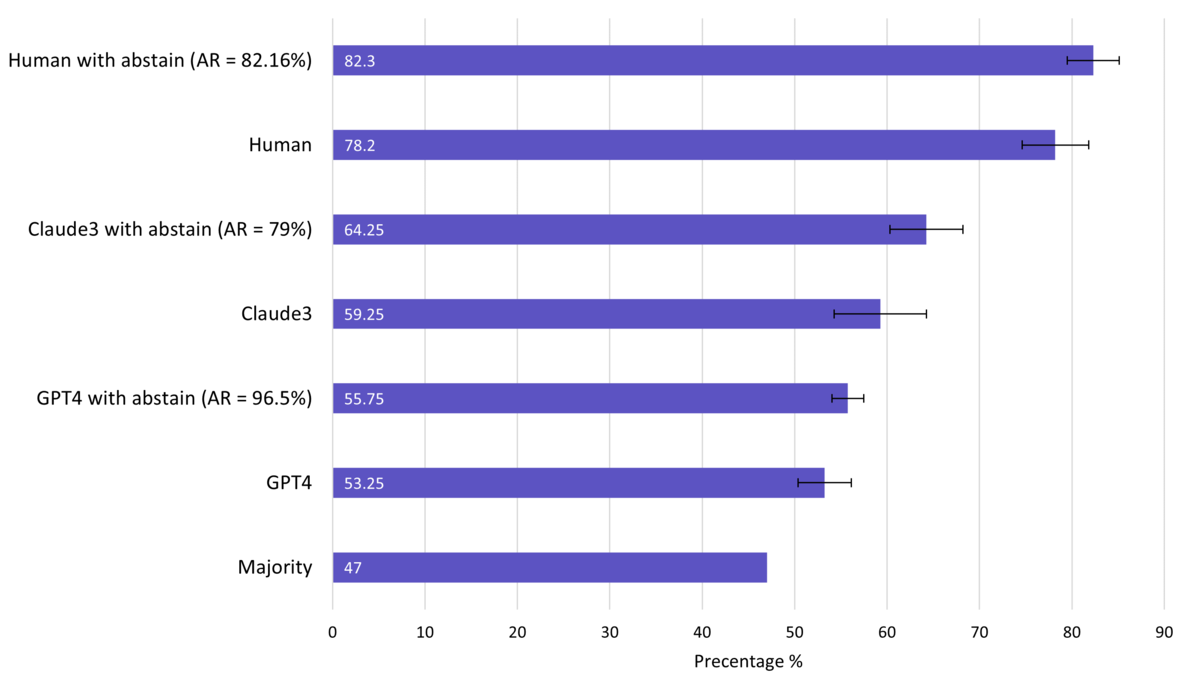

Supplement: Multimedia Appendix 12 [file jmir-v27-e64452-s012.png]
