# Supplementary material for: Performance of Large Language Models in Numerical Versus Semantic Medical Knowledge: Cross-Sectional Benchmarking Study on Evidence-Based Questions and Answers
Source: J Med Internet Res. 2025 Jul 14;27:e64452. doi: 10.2196/64452 (PMC12279315; doi:10.2196/64452)
Supplement: Multimedia Appendix 5 [file jmir-v27-e64452-s005.docx]

### Table S2 - Distributions of answers according to question type

| Question type | Median | First quartile | Third quartile | Mean | SD |
| --- | --- | --- | --- | --- | --- |
| Semantic | 77 | 73 | 81 | 77.72 | 6.074 |
| Risk Factor | 81 | 80 | 84 | 82.37 | 3.635 |
| Association | 68 | 66 | 71 | 68.82 | 4.29 |
| Sensitivity | 54 | 52 | 58 | 55.488 | 4.19 |
| PLR | 69 | 66 | 73 | 69.535 | 4.854 |
| Incidence | 66 | 63.5 | 68 | 66.028 | 3.21 |
| Prevalence | 58 | 56 | 60 | 58.437 | 3.60 |
| Associated Risk | 72 | 70.5 | 74 | 72.52 | 2.97 |
| Relative Risk | 71 | 69 | 72.5 | 71.42 | 3.47 |
| NLR | 69 | 66 | 73 | 69.827 | 5.23 |
| Specificity | 60 | 55 | 64 | 59.67 | 5.88 |
| PPV | 62 | 59 | 66.25 | 63.51 | 5.16 |
| NPV | 62 | 58 | 64 | 62.145 | 5.08 |
