# Supplementary material for: Performance of Large Language Models in Numerical Versus Semantic Medical Knowledge: Cross-Sectional Benchmarking Study on Evidence-Based Questions and Answers
Source: J Med Internet Res. 2025 Jul 14;27:e64452. doi: 10.2196/64452 (PMC12279315; doi:10.2196/64452)
Supplement: Multimedia Appendix 9 [file jmir-v27-e64452-s009.docx]

### Table S3- Human and prompt validation- significant comparison:

|  | Majority | Claude3 without I do not know | Claude3 with I do not know | GPT4 without I do not know | GPT4 with I do not know | Human without I do not know | Human with I do not know |
| --- | --- | --- | --- | --- | --- | --- | --- |
| Claude3 without I do not know | *P = .0163* | *NA* | *P = .1698* | *P = .105* | *P = .2607* | *P=.0001* | *P<.0001* |
| Claude3 with I do not know | *P = .003* | *P =.1698* | *NA* | *P = .006* | *P = .02* | *P=.0004* | *P<.0001* |
| GPT4 without I do not know | *P = .0205* | *P = .105* | *P = .006* | *NA* | *P = .24* | *P<.0001* | *P<.0001* |
| GPT4 with I do not know | *P = .002* | *P = .2607* | *P = .02* | *P = .24* | *NA* | *P<.0001* | *P<.0001* |
