# Supplementary material for: Performance of Large Language Models in Numerical Versus Semantic Medical Knowledge: Cross-Sectional Benchmarking Study on Evidence-Based Questions and Answers
Source: J Med Internet Res. 2025 Jul 14;27:e64452. doi: 10.2196/64452 (PMC12279315; doi:10.2196/64452)
Supplement: Multimedia Appendix 11 [file jmir-v27-e64452-s011.docx]

#### Table S4- Proportion comparison according to sub labels:

| 95% confidence interval | Proportion p-value | GPT4 Accuracy | Claude3 Accuracy | Sub label | Label |
| --- | --- | --- | --- | --- | --- |
| [-0.19 - 0.15] | <.0001 | 63.97% (4438/ 6938) | 47.05% (2060/ 4378) | 1^st^ Answer | Answer distribution |
| [0.32 - 0.35] | <.0001 | 47.86% (4141/ 8653) | 80.96% (4546/ 5615) | 2^nd^ Answer | Answer distribution |
| [-0.30 - 0.25] | <.0001 | 64.36% (1929/ 2997) | 36.85% (667/ 1810) | 3^rd^ Answer | Answer distribution |
| [-0.04 - 0.04] | .97 | 60.35% (1076/ 1783) | 60.2% (717/ 1191) | Cardiovascular System | Medical discipline |
| [-0.01 - 0.05] | .27 | 58.43% (1306/ 2235) | 60.33% (844/ 1399) | Digestive System | Medical discipline |
| [-0.05 - 0.06] | .96 | 59.03% (428/ 725) | 59.36% (298/ 502) | Endocrine System | Medical discipline |
| [0.004 - 0.07] | .03 | 57.89% (1207/ 2085) | 61.7% (862/ 1397) | Infectious Disease | Medical discipline |
| [0.11 - 0.19] | <.0001 | 53.5% (704/ 1316) | 68.7% (676/ 984) | Neoplastic Disease | Medical discipline |
| [0.01 - 0.09] | .01 | 55.54% (938/ 1689) | 60.43% (681/ 1127) | Nervous System | Medical discipline |
| [-0.02 - 0.07] | .21 | 56.29% (720/ 1279) | 59.17% (484/ 818) | Respiratory System | Medical discipline |
| [-0.008 - 0.08] | .11 | 54.1% (679/ 1255) | 57.78% (464/ 803) | Genitourinary System | Medical discipline |
| [0.01 - 0.05] | .0004 | 56.88% (4232/ 7440) | 60.06% (3181/ 5296) | Disorders | Medical subject |
| [-0.003 - 0.08] | .07 | 60.43% (1017/ 1683) | 64.39% (463/ 719) | Imaging and Procedures | Medical subject |
| [0.07 - 0.14] | <.0001 | 53.45% (1008/ 1886) | 64.08% (619/ 966) | Lab tests | Medical subject |
| [0.05 - 0.09] | <.0001 | 56.05% (3438/ 6134) | 63.08% (2454/ 3890) | Symptom and Signs | Medical subject |
| [0.0006 - 0.03] | .04 | 58.73% (4842/ 8245) | 60.46% (3410/ 5640) | High | Prevalence |
| [0.07 - 0.15] | <.0001 | 53.21% (871/ 1637) | 64.37% (598/ 929) | Low | Prevalence |
| [0.05 - 0.1] | <.0001 | 56.2% (2919/ 5194) | 63.83% (2125/ 3329) | Med | Prevalence |
| [0.02 - 0.05] | <.0001 | 54.54% (4418/ 8100) | 58.25% (3378/ 5799) | Long | QA length |
| [0.04 - 0.07] | <.0001 | 57.53% (6169/ 10723) | 62.74% (4098/ 6532) | Medium | QA length |
| [0.02 - 0.08] | .0004 | 60.66% (1451/ 2392) | 66.13% (1107/ 1674) | Short | QA length |
| [0.02 - 0.09] | .0007 | 49.5% (1146/ 2315) | 55.04% (906/ 1646) | Association | QA Type |
| [-0.03 - 0.05] | .62 | 53.86% (802/ 1489) | 54.88% (720/ 1312) | Incidence | QA Type |
| [-0.02 - 0.04] | .5 | 58.25% (1391/ 2388) | 59.28% (1268/ 2139) | Risk Factor | QA Type |
| [0.05 - 0.07] | <.0001 | 58.11% (8394/ 14446) | 63.99% (5510/ 8611) | Sensitivity | QA Type |
